# Development of quality control standards for radiation therapy equipment in Canada

**DOI:** 10.1120/jacmp.v8i1.2380

**Published:** 2007-02-28

**Authors:** Peter Dunscombe, Harry Johnson, Clement Arsenault, George Mawko, Jean‐Pierre Bissonnette, Jan Seuntjens

**Affiliations:** ^1^ University of Calgary/Tom Baker Cancer Centre Calgary Alberta Canada; ^2^ CancerCare Manitoba Winnipeg Manitoba Canada; ^3^ Hôpital Dr Georges–L. Dumont Centre d'Oncologie Dr Léon–Richard Moncton New Brunswick Canada; ^4^ QE II Health Sciences Centre Diagnostic Imaging Department Halifax Nova Scotia Canada; ^5^ Princess Margaret Hospital Radiation Physics Department Toronto Ontario Canada; ^6^ McGill University Health Centre Medical Physics Unit Montreal Quebec Canada

**Keywords:** radiotherapy, quality control, Canada

## Abstract

Among the essential components of a comprehensive quality assurance program in radiotherapy are the quality control protocols to be used on the equipment and, in particular, the performance objectives and criteria. In the present work, we describe the development of a suite of quality control documents for use across Canada. Following a generic format, we are generating concise, clear standards for the most commonly used equipment in radiotherapy, with the emphasis on performance measures. The final standards of performance are confirmed following cross‐country consultation facilitated by the availability of draft documents on the Canadian Medical Physics web site.

PACS number: 87.53.Xd

## I. INTRODUCTION

The provision of health care services to Canadians is largely the responsibility of the ten provinces and three territories. Although the services that must be provided free to the population are specified in the federal *Canada Health Act*, operational and financial aspects of service provision are determined by the provinces and territories. This service delivery structure applies equally to cancer care as it does to other medical services.

The Canadian Association of Provincial Cancer Agencies (CAPCA) is a body that meets regularly to discuss issues of common interest to the organizations responsible for the delivery of cancer care in Canada. A proposal recently accepted by CAPCA was to initiate a process aimed at harmonizing quality assurance activities in radiation treatment programs across the country. This initiative has resulted in a draft document titled *Standards for Quality Assurance at Canadian Radiation Treatment Centres*. (hereinafter *Standards*). Practical and essential components of any quality assurance program for radiation therapy are the quality control tests carried out on the increasingly sophisticated equipment used in the planning and delivery of treatment. The draft document referred to appendices, which, when developed, would specify the performance standards to be required of equipment used in the preparation and delivery of radiation therapy to all Canadian cancer patients.

The development of the quality control standards themselves was, appropriately, delegated to the national professional body representing Canadian radiation oncology physicists: the Canadian Organization of Medical Physicists (COMP). In turn, COMP established a Task Group, the members of which are the authors of the present work, to coordinate the generation of the standards documents.

We here describe the philosophy, format, and process adopted by the Task Group, and we refer readers to the web site on which both the approved and draft standards may be reviewed.

## II. MATERIALS AND METHODS

### A. Documents

The documents upon which the standards are based originated from several sources. Some of the original documents were developed by the Medical Physics Professional Advisory Committee of Cancer Care Ontario and its predecessor, the Ontario Cancer Treatment and Research Foundation. Documents dealing with more recent technology were either specifically commissioned by CAPCA for the purpose of standards development, or, in one case, was based on a recent publication. Also vital to the present project are the many publications relating to quality control and quality assurance in radiotherapy. These include—but are not limited to—recommendations promulgated by the American Association of Physicists in Medicine,^(1)^ The Institute of Physics and Engineering in Medicine,^(2)^ and medical physics compendia.^(^ ^3^ ^,^ ^4^ ^)^

### B. Philosophy and scope

The philosophy behind the development of the *Standards* documents was that they should focus on the standards themselves and not include descriptions of how the tests are performed. It is assumed that physicists who perform or who supervise the performance of the tests possess an appropriate level of knowledge. Otherwise, the bibliography refers the physicist to the recent literature on the subject. Furthermore, radiation safety has not been specifically included. To do so would require updating the documents each time federal or provincial regulations change, and the Task Group did not feel able to accept this responsibility. However, for completeness, some of the more straightforward tests performed on a daily basis were included.

The *Standards* documents are intended to be brief and unambiguous. Distribution through a web site facilitates updates as experience with new techniques is gained.

To maintain focus and unambiguity, a generic document format was adopted, with these sections:

- Introduction—largely generic
- Performance Objectives and Criteria—generic
- System Description—custom
- Acceptance Tests and Commissioning—largely generic
- Quality Control of Equipment—largely generic
- Documentation—generic
- Table of QC Tests—custom entries in a generic format
- References and Bibliography—custom

### C.1 Performance Objectives and Criteria

The generic Performance Objectives and Criteria section includes six classes:

- Functionality
- Reproducibility
- Accuracy
- Characterisation and Documentation
- Data Transfer and Validation
- Completeness

As an example of a generic portion of the documents, Appendix 1 shows the exact wording used in the Performance Objectives and Criteria section. The attempt here, and elsewhere in the generic sections, is to be unambiguous and, where appropriate, prescriptive. The six classes were considered to encompass the range of responses that adequately describe the results of testing. Frequency of testing is also clearly specified, but provides flexibility for operational considerations.

### D. Document generation and review

Regardless of whether a source document was commissioned specifically for the development of the *Standards* or had been generated before this project was initiated, it was sent to a knowledgeable Canadian medical physicist for external review. The reviewer looked at the source document in the light of relevant international recommendations and provided detailed comments on the suggested standards.

Two of the authors of the present work were assigned to each document, one as the primary task group reviewer and one as the secondary reviewer. It was the responsibility of these two members of the group to consider the source document, the external reviewer's comments, and the international literature; to recommend the draft standards; and then to prepare the relevant documentation in the format described above. This task has been simplified for the more recent documents, because the generic format had been decided, and standards could be commissioned to be consistent with that format.

Once the primary and secondary task group reviewers had agreed on their version of the standard, that standard was circulated to whole Task Group for approval. Following this final internal step, the standard was posted on www.medphys.ca for consideration by the Canadian medical physics community at large.

During the next phase, which is ongoing, the comments from physicists “in the field” are being solicited and considered. Comments are fed back into the internal review process, and the standard is modified if required. Comments received so far have ranged from technical to language to typographical. Once the Task Group has reviewed, incorporated, and approved suggested changes, the standards undergo one final formal review by the Canadian Organization of Medical Physicists before national adoption. So far, the national review process has been completed for the first six standards.

## III. RESULTS

At the time of writing, standards documents for the following equipment have been approved by COMP:

- Linear accelerators
- Conventional simulators
- Orthovoltage units
- Cobalt units
- Multileaf collimators
- Electronic portal imaging devices

The following draft standards have been posted and are currently under national review:

- Remote afterloading brachytherapy equipment
- Major dosimetry equipment
- CT simulators
- Prostate brachytherapy equipment
- SRS/T equipment.

Tables 1–6 show the six currently approved standards and illustrate the generic format adopted. Notes (not shown for space reasons) accompany each table to clarify the meaning of numerical tolerances and action levels, but these notes do not recommend measurement techniques.

- Standards currently under development include those for
- Data management systems
- Treatment planning systems
- Intensity‐modulated radiation therapy

**Table 1:** Quality control tests for medical linear accelerators (tolerances and action levels are specified in millimeters unless otherwise stated)

Designator	Test	Performance	
		Tolerance	Action
**Daily**			
DL1	Door interlock/last person out	Functional	
DL2	Motion interlock	Functional	
DL3	Couch brakes	Functional	
DL4	Beam status indicators	Functional	
DL5	Patient audiovisual monitors	Functional	
DL6	Room radiation monitors	Functional	
DL7	Beam interrupt/counters	Functional	
DL8	Lasers/crosswires	1	2
DL9	Optical distance indicator	1	2
DL10	Optical back pointer	2	3
DL11	Field size indicator	1	2
DL12	Output constancy—photons	2%	3%
DL13	Dynamic wedge factors	1%	2%
DL14	Output constancy—electrons	2%	3%
**Monthly**			
ML1	Emergency off	Functional	
ML2	Wedge, tray cone interlocks	Functional	
ML3	Accessories integrity and centering	Functional	
ML4	Gantry angle readouts	0.5°	1°
ML5	Collimator angle readouts	0.5°	1°
ML6	Couch position readouts	1	2
ML7	Couch isocenter	1	2
ML8	Couch angle	0.5°	1°
ML9	Optical distance indicator	1	2
ML10	Crosswire centering	1	2
ML11	Light/radiation coincidence	1	2
ML12	Field size indicator	1	2
ML13	Relative dosimetry	1%	2%
ML14	Central axis depth dose reproducibility	1 (2%)	2 (3%)
ML15	Beam flatness	2%	3%
ML16	Beam symmetry	2%	3%
ML17	Records	Complete	
**Annually**			
AL1	Reference dosimetry—TG51	1%	2%
AL2	Relative output factor reproducibility	1%	2%
AL3	Wedge transmission factor reproducibility	1%	2%
AL4	Accessory transmission factor reproducibility	1%	2%
AL5	Output reproducibility vs. gantry angle	1%	2%
AL6	Beam symmetry reproducibility vs. gantry angle	2%	3%
AL7	Monitor chamber linearity	1%	2%
AL8	End monitor effect	0.1 MU	0.2 MU
AL9	Collimator rotation isocenter	1	2
AL10	Gantry rotation isocenter	1	2
AL11	Couch rotation isocenter	1	2
AL12	Coincidence of collimator, gantry, couch axes	1	2
AL13	Coincidence of isocenters	1	2
AL14	Couch deflection	3	5
AL15	Independent quality control review	Complete	

**Table 2:** Quality control tests for conventional simulators (tolerances and action levels are specified in millimeters unless otherwise stated)

Designator	Test	Performance	
		Tolerance	Action
**Daily**			
DS1	Door interlock	Functional	
DS2	Motion interlock	Functional	
DS3	Beam status indicators	Functional	
DS4	Emergency off buttons	Functional	
DS5	Collision avoidance	Functional	
DS6	Lasers/crosswires	1	2
DS7	Optical distance indicator	1	2
DS8	Crosswires/reticle/block tray	1	2
DS9	Light/radiation coincidence	1	2
DS10	Field size indicators	1	2
**Monthly**			
MS1	Gantry angle readouts	0.5°	1°
MS2	Collimator angle readouts	0.5°	1°
MS3	Couch position readouts	1	2
MS4	Alignment of FAD movement	1	2
MS5	Couch isocenter	2	3
MS6	Couch parallelism	1	2
MS7	Laser/crosswire isocentricity	1	2
MS8	Optical distance indicator	1	2
MS9	Crosswire centering	1	2
MS10	Light/radiation coincidence	1	2
MS11	Field size indicators	1	2
MS12	Records	Complete	
**Six‐monthly**			
SS1	Lead apron	Functional	
SS2	${\text{kV}}_{p}$	5%	10%
SS3	Reference dosimetry	5%	10%
SS4	Beam quality (HVL)	5%	10%
SS5	Automatic exposure control	5%	10%
SS6	Focal spot	Reproducible	
SS7	Contrast	Reproducible	
SS8	Resolution	Reproducible	
SS9	Fluoroscopic timer	5%	10%
**Annually**			
AS1	Redefine isocenter	1	2
AS2	Couch deflection	3	5
AS3	Alignment of focal spots	0.5	1
AS4	Independent quality control review	Complete	

**Table 3:** Quality control tests for kilovoltage radiotherapy units (tolerances and action levels are specified in millimeters unless otherwise stated)

Designator	Test	Performance	
		Tolerance	Action
**Daily**		
DK1	Patient monitoring audiovisual devices	Functional	
DK2	Door closing mechanism and interlock	Functional	
DK3	Couch movement and brakes	Functional	
DK4	Unit motions and motion stops	Functional	
DK5	Interlocks for added filters/kV‐filter choice	Functional	
DK6	Beam status indicators	Functional	
DK7	Beam‐off at key‐off test	Functional	
DK8	Emergency off test	Functional	
DK9	kV and mA indicators	Functional	
DK10	Backup timer/monitor unit channel check	1%	2%
DK11	Dosimetric test: output check	3%	5%
**Monthly**		
MK1	Mechanical stability and safety	Functional	
MK2	Cone selection and competency	Functional	
MK3	Physical distance indicators	2	3
MK4	Accuracy of head tilt and rotation readouts	1°	1.5°
MK5	Light/x‐ray field coincidence	2	3
MK6	Light field size	2	3
MK7	X‐ray field size indicator	2	3
MK8	X‐ray field uniformity/filter integrity	5%	8%
MK9	Timer and end effect error	Characterize	±0.05 min
MK10	Output linearity	1%
MK11	Output reproducibility	Characterize	<.03 CoV
MK12	Beam quality	10%	15%
MK13	Output calibration verification	2%	3%
MK14	Timer accuracy verification	2%	3%
MK15	Records	Complete	
**Annually**		
AK1	Reference dosimetry	1%	2%
AK2	Alignment of focal spots	0.5	1
AK3	kVp measurement	5%	10%
AK4	Focal spot size	Reproducible	
AK5	Independent quality control review	Complete	

**Table 4:** Quality control tests for C60o teletherapy units (tolerances and action levels are specified in millimeters unless otherwise stated)

Designator	Test	Performance	
		Tolerance	Action
**Daily**		
DCO1	Door interlock/last person out	Functional	
DCO2	Motion interlock	Functional	
DCO3	Couch brakes	Functional	
DCO4	Beam status indicators	Functional	
DCO5	Patient audiovisual monitors	Functional	
DCO6	Room radiation monitors	Functional	
DCO7	Emergency off	Functional	
DCO8	Beam interrupt/counters	Functional	
DCO9	Head swivel lock	Functional	
DCO10	Lasers/crosswires	1	2
DCO11	Optical distance indicator	1	2
DCO12	Optical back pointer	2	3
DCO13	Field size indicator	1	2
**Monthly**		
MCO1	Latching of wedges, trays	Functional	
MCO2	Wedge interlocks	Functional	
MCO3	Gantry angle readouts	0.5°	1°
MCO4	Collimator angle readouts	0.5°	1°
MCO5	Couch position readouts	1	2
MCO6	Couch rotation isocenter	2	3
MCO7	Optical distance indicator	1	2
MCO8	Crosswire centering	1	2
MCO9	Light/Radiation coincidence	2	3
MCO10	Field size indicator	1	2
MCO11	Relative dosimetry	1%	2%
MCO12	Shutter error	Reproducible	
MCO13	Beam symmetry (source position)	2%	3%
MCO14	Records	Complete	
**Annually**		
ACO1	Reference dosimetry	1%	2%
ACO2	Relative output factor reproducibility	1%	2%
ACO3	Central axis depth dose reproducibility	1%	2%
ACO4	Wedge transmission factor reproducibility	1%	2%
ACO5	Accessory transmission factor reproducibility	1%	2%
ACO6	Output reproducibility vs. gantry angle	1%	2%
ACO7	Beam symmetry reproducibility vs. gantry angle	2%	3%
ACO8	Timer linearity	1%	2%
ACO9	Shutter error	0.03 min.	0.05 min.
ACO10	Collimator rotation isocenter	2	3
ACO11	Gantry rotation isocenter	2	3
ACO12	Couch rotation isocenter	2	3
ACO13	Coincidence of collimator, gantry, couch axes	2	3
ACO14	Coincidence of isocenters	2	3
ACO15	Couch deflection	3	5
ACO16	Independent quality control review	Complete	

**Table 5:** Quality control tests for multileaf collimators (tolerances and action levels are specified in millimeters unless otherwise stated)

Designator	Test	Performance	
		Tolerance	Action
**Patient‐specific**		
PM1	Verification of transferred data vs. printed template	1	2
PM2	Daily verification of correct data	Reproducibility	
PM3	Verification of record and verify programming	Reproducibility	
**Monthly**		
MM1	Digitizer check (if used)	Functional	
MM2	Light and radiation field coincidence	1	2
MM3	Leaf positions for standard field template	1	2
MM4	Electron field interlocks	Functional	
MM5^a^	Leaf alignment	1
MM6	Records	Complete	
Yearly		
AM1	Leaf transmission (all energies)	Reproducibility	
AM2	Leakage between leaves (all energies)	Reproducibility	
AM3^a^	Transmission through abutting leaves	Reproducibility	
AM4	Stability with gantry rotation	Reproducibility	
AM5	Alignment with jaws	1
AM6	Independent quality control review	Complete	

a May not apply to all designs.

**Table 6:** Quality control tests for electronic portal imaging devices (tolerances and action levels are specified in millimeters unless otherwise stated)

Designator	Test	Performance	
		Tolerance	Action
**Daily**		
DE1	Mechanical integrity	Functional	
DE2	Electrical integrity	Functional	
DE3	Collision interlocks	Functional	
DE4	Image quality	Reproducibility	
**Monthly**		
ME1	Positioning in the imaging plane	1	2
ME2	Positioning perpendicular to the imaging plane	10	20
ME3	Image quality	Reproducibility	
ME4	Artifacts	Reproducibility	
ME5	Spatial distortion	1	2
ME6	Monitor controls	Reproducibility	
ME7	Records	Complete	
**Six monthly**		
SE1	Spatial resolution	Reproducibility	
SE2	Noise	Reproducibility	
SE3	On‐screen measurement tools	0.5	1
SE4	Setup verification tools	0.5 (0.5°)	1 (1°)
**Annually**		
AE1	Independent quality control review	Complete	

These latter standards will be posted as they become available. The interested reader is directed to www.medphys.ca to review the complete results of the project to date.

## IV. DISCUSSION AND CONCLUSIONS

This project has achieved its objectives to date. The largely generic format of the *Standards* has aided clarity of interpretation and expedited development of the documents—particularly the later documents, which could be composed to fit the format. At some stage in the future, if it is deemed desirable, all the available documents could easily be consolidated into one because so much of the content is generic.

Posting the drafts on an easily accessible web site facilitates feedback and constitutes a method for obtaining a national consensus on the standards. The medical physics community can consider not only the objectives and criteria of the tests, but also the resource implications of adopting the standards. Furthermore, standards approved at this time may easily be updated as new knowledge and equipment become available. Updates can be disseminated almost instantaneously.

The structure of health care delivery in Canada is not conducive to the development of nationally legislated quality control standards, and such legislation is unlikely to be passed in this case. However, once approved and adopted, the standards discussed here may well form an easily monitored component of licensing and accreditation activities applied to cancer treatment facilities.

## Appendix

Objectives and criteria for the evaluation of the performance of radiotherapy equipment fall into several categories:

1. Functionality. Equipment systems and sub‐systems for which the criterion of performance is “Functional” are either working correctly or not. Such systems are commonly associated with the safety features of the equipment or installation. Operating a facility which has failed a test of functionality has the potential to expose patients and staff to hazardous conditions.
2. Reproducibility. The results of routine quality control tests, for which reproducibility is the criterion, are assessed against the results obtained at installation from the accepted unit. Tolerances and action levels may be set for parameters that can be quantified.
3. Accuracy. Accuracy is the deviation of the measured value of a parameter from its expected or defined value. An example is template positional accuracy.
4. Characterisation and documentation. In some cases it is necessary to make measurements to characterise the performance of a piece of equipment before it can be used clinically. An example is the measurement of the ion collection efficiency of an ionization chamber.
5. Data transfer and validation. Many systems in use in radiation therapy, and elsewhere, rely heavily upon the appropriate data, such as prescription point and wedge orientation, being input and accurately transmitted through the systems. This category of test is intended to confirm that these processes, involving both humans and machines, are being correctly performed.
6. Completeness. The use of this term is restricted to the periodic review of quality control procedures, analysis and documentation.

For quantities that can be measured, tolerance and action levels may be defined.

- Tolerance Level. For a performance parameter that can be measured, a tolerance level is defined. If the difference between the measured value and its expected or defined value is at or below the stated tolerance level then no further action is required as regards that performance parameter.
- Action Level. If the difference between the measured value and its expected or defined value exceeds the action level then a response is required immediately. The ideal response is to bring the system back to a state of functioning that meets all tolerance levels. If this is not immediately possible, then the use of the equipment must be restricted to clinical situations in which the identified inadequate performance is of no or acceptable and understood clinical significance. The decision concerning the most appropriate response is made by the supervising physicist in conjunction with the users of the equipment and others as appropriate. If the difference between the measured value and its expected or defined value lies between the tolerance and action levels, several courses of action are open. For a problem that is easily and quickly rectifiable, remedial action should be taken at once. An alternative course of action is to delay remedial action until the next scheduled maintenance period. Finally, the decision may be made to monitor the performance of the parameter in question over a period of time and to postpone a decision until the behavior of the parameter is confirmed. Once again, this will be a decision made by the supervising physicist in consultation with the users of the equipment and others as appropriate.

Documentation of equipment performance is essential and is discussed later. However, at the conclusion of a series of quality control tests it is essential to inform the users of the equipment of its status. If performance is within tolerance verbal communication with the users is sufficient. If one or more parameters fail to meet Action Level criteria, and immediate remedial action is not possible, then the users of the equipment must be informed in writing of the conditions under which the equipment may be used. Compliance with Action Levels but failure to meet Tolerance Levels for one or more parameters may be communicated verbally or in writing depending on the parameters and personnel involved. The judgment of those involved will be required to make this decision.
